# Infant-feeding practices and infant survival by familial wealth in London, 1752–1812

**DOI:** 10.1080/1081602X.2019.1580601

**Published:** 2019-03-07

**Authors:** Romola Jane Davenport

**Affiliations:** Cambridge Group for the History of Population and Social Structure, Department of Geography, University of Cambridge, Cambridge, UK

**Keywords:** Infant mortality, wet-nursing, breastfeeding, urban mortality, smallpox

## Abstract

Anecdotal evidence indicates that high-status women in England generally did not breastfeed their children in the seventeenth and early eighteenth centuries. Metropolitan families of varied social status also often sent their children out of London for wet-nursing. However, anecdotal sources and rural burial registers also suggest that these practices declined rapidly from the mid-eighteenth century, and were replaced by a culture of maternal breastfeeding in all social classes. These changes in infant-feeding practices have been argued to explain much of the dramatic improvement in infant mortality rates in London in this period. Here we used quantitative evidence from a partial family reconstitution of the London parish of St. Martin in the Fields to test these claims. Using birth interval analysis to infer breastfeeding patterns in families by four categories of wealth, we found that birth intervals were close to the national average in pauper and poor families, but much shorter in wealthier families, in the period 1752–74. We also found evidence that many infants especially in wealthier families were missing from observation, consistent with high levels of rural wet-nursing. Both these phenomena declined between 1775 and 1812, suggesting a convergence in breastfeeding practices to the national norm. We used event history analysis, with corrections to aggregate rates for missing infants, to compare mortality rates over time and by wealth category. We found that infant mortality was initially higher in wealthier families, but declined in all groups over the period 1752–1812. We conclude that increases in maternal breastfeeding were probably important in improving survival of infants from wealthier families, however changes in breastfeeding patterns were insufficient to account for the ubiquitous improvements in mortality of urban-born infants in this period.

## 1. Introduction

This paper addresses the early origins of two key features of modern demographic regimes: the disappearance of the ‘urban penalty’, and the ubiquity of socioeconomic gradients in life expectancy. We present evidence regarding the earliest phase of urban mortality decline in England, derived from analysis of individual-level data from the London parish of St. Martin in the Fields in the period 1752–1812. In contrast to previous studies of English populations our data include information on familial socioeconomic status across the social spectrum of the parish, making it possible to investigate levels and trends in mortality amongst different sub-groups within the parish. This introduction reviews the evidence for patterns of mortality decline in London compared with the English population as a whole, and for socioeconomic differentials in levels and trends of infant mortality in this period.

The dramatic decline in urban mortality across north-western Europe in the period 1750–1820 constitutes a major outstanding puzzle in historical demography. In the seventeenth and early eighteenth - centuries European cities are argued to have operated as ‘urban graveyards’, with death rates so high as to require a net flow of in-migrants even to maintain their population size. Wrigley famously estimated that half the natural growth of the English population was consumed by London’s mortality regime in this period (Wrigley, 1967). Jan de Vries has argued that excessive urban mortality rates precluded modern economic growth, with its concomitant rapid urbanisation, because no national population could produce a rural population surplus sufficient to maintain a very large urban component (de Vries, 1984, Chapter 10). However, in the second half of the eighteenth century a dramatic change occurred, and baptisms began to exceed burials from around 1770 in a large number of towns and cities in north-western Europe. Indeed it is likely that much of the relatively modest improvement in life expectancy over the eighteenth century in the English population is attributable to improvements in mortality in urban populations (Wrigley, Davies, Oeppen, & Schofield, 1997, p. 274; Galley & Shelton, 2001), in contrast to the braking function of urban death rates on life expectancy in both the seventeenth and nineteenth centuries (Woods, 1985, 2000, Chapter 9; Wrigley & Schofield, 1989, pp. 415–416).

**Figure 1.**
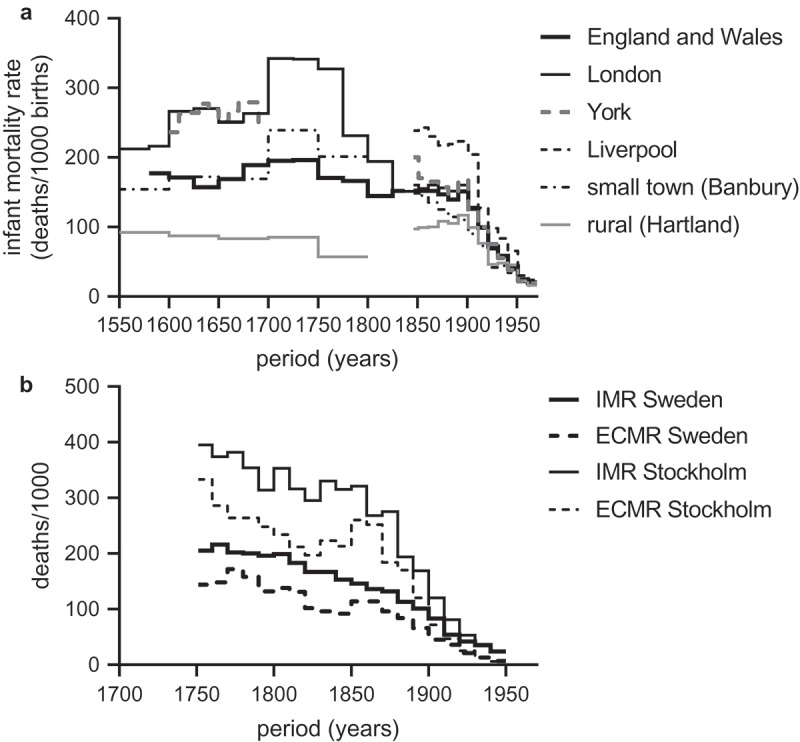
Long-run patterns in urban mortality rates amongst infants in England (a) and infants and young children in Sweden (b). *Notes*: IMR refers to deaths in the first year of life per 1,000 live births; ECMR refers to deaths at ages 1–4 years per 1,000 children aged 1–4 years.*Sources*: England and Wales: Wrigley et al., 1997; Human Mortality Database. London: Landers, 1993, p. 136; *Annual Reports of the Registrar-General of births, deaths, and marriages in England*, and decennial supplements. Liverpool: Annual reports of the Registrar-General and decennial supplements; York: Galley, 1998, Table 4.9; Annual reports of the Registrar-General and decennial supplements. Banbury: Wrigley & Schofield, 1983; Annual reports of the Registrar-General and decennial supplements; Sweden: Woods, 1993, Appendix Table 2.

Our understanding of pre-nineteenth-century urban mortality levels and trends rests, however, on a very fragile empirical base. In most cases the only evidence we have for reductions in urban mortality are rises in the ratios of baptisms to burials, and these are open to multiple interpretations (Sharlin, 1978; van der Woude, 1982). Very few urban populations collected the data required to calculate age-specific mortality rates for the population as a whole, and even where individual-level data are available the high mobility and diversity of urban populations make them particularly intractable to robust historical demographic methodologies (Davenport, 2016; Galley, 1995). In the case of England, before the early nineteenth century there are few census-type data giving the age and sex structure, or even the size, of urban populations, and the larger urban populations were characterised by markets for burials and diverse registration practices that seriously compromise measures based on Anglican parish registers (Boulton, 2014; Galley, 1998; Landers, 1993). The only robust evidence that we have currently for English towns in the century before the introduction of civil registration in 1837 derive from several small market towns (with populations of 2,000–3,000), and from London Quakers (Figure 1(a)). Quakers comprised a relatively affluent and unrepresentative component of the metropolitan population. However, estimates of infant mortality in the Quaker sample were reassuringly similar to those derived by cruder methods from the London bills of mortality, falling from 342 deaths per thousand births in the period 1700–49 to 151/1000 by the second quarter of the nineteenth century (Landers, 1993, p. 136). Similar trends are evident in Stockholm over the same period (Figure 1(b)).

Despite its importance the causes of the dramatic falls in urban infant mortality evident in Figure 1 remain unclear. At the aggregate national - level neonates (infants in the first month of life) experienced the most significant improvements in survival (from a peak of 110 deaths per thousand births in the period 1675–99 to 57/1000 by 1800–24), and this accounted for almost all the observed reduction in infant mortality in the English population in the eighteenth and early nineteenth centuries (Wrigley et al., 1997, pp. 236–237). In Landers’ London Quaker sample improvements were also greatest in the first three months of life, and neonatal mortality fell from 125/1000 in the first quarter of the eighteenth century to 40/1000 by the first quarter of the nineteenth (Landers, 1993, p. 136). In smaller towns rates of neonatal mortality were also initially much higher than rural populations, but appear to have converged across settlement types by the early nineteenth century (Galley & Shelton, 2001; Smith & Oeppen, 2006; Wrigley et al., 1997, pp. 228–237, 276–279). However in contrast to national trends, where there was no improvement in mortality at ages three months to two years over the period 1700–1837, London Quakers experienced significant gains in survival at these ages as well.

Landers demonstrated that smallpox was a major driver of mortality trends in Quaker children aged between one and 10 years of age. However, he was unable to identify the causes of improvements in infancy, when smallpox was a relatively minor cause of death. He hypothesised that the very high rates of neonatal mortality amongst London Quakers in the seventeenth and early eighteenth centuries were caused in the main by gastrointestinal infections associated with artificial feeding, and thus that the dramatic improvement in neonatal mortality after 1750 was probably due to a decline in artificial feeding (Landers, 1993, p. 152). Evidence for artificial feeding of Quaker neonates came from two sources. First, birth intervals were very short in London Quaker families before 1750, consistent with limited or no maternal breastfeeding. Second, Quaker neonatal mortality displayed a strong summer peak, consistent with hand-feeding of infants and consequent exposure to contaminated food. Birth intervals amongst London Quakers lengthened after 1750, consistent with increasing maternal breastfeeding (possibly as a means of limiting marital fertility) (Landers, 1990). Curiously, however, the summer peak in neonatal mortality persisted, despite large improvements in neonatal mortality. Landers accounted for this pattern by the persistence of hand-feeding in a minority of infants, amongst whom mortality became concentrated. He also acknowledged the possibility that the summer peak reflected a tradition of feeding infants ‘purges’ during the first few days of life, in the belief that the maternal colostrum secreted during the first few days after birth was unsuitable for infants (Fildes, 1986, Chapter 2; Landers, 1993, pp. 141–149).

Landers’ evidence for limited maternal breastfeeding amongst London Quakers is consistent with other sparse quantitative evidence for urban and elite infant-feeding practices in early modern England. Wilson and Wrigley and colleagues argued that breastfeeding was relatively ubiquitous and of long duration in the English non-metropolitan population in the early modern period. However in London Finlay reported very short birth intervals in two wealthy intramural parishes in the period 1580–1650, and contrasted these with the longer birth intervals of two poorer metropolitan parishes (Finlay, 1981, pp. 142–146). Galley reported very short birth intervals in wealthy parishes in seventeenth century York (and a summer peak in neonatal mortality consistent with very early weaning) (Galley, 1998, pp. 101–103, 177–121). Birth intervals were also of below average duration in the market towns compared with rural parishes within the national Cambridge Group reconstitution sample (Wilson, 1986).

In addition to these rather meagre quantitative data there is substantial documentary evidence from diaries and letters, medical accounts and advice manuals, that elite English women in the seventeenth and early eighteenth centuries generally did not breastfeed their own children (Campbell, 1989; Fildes, 1986, 1988a). This literature indicates that the main alternative to maternal breastfeeding was wet-nursing, although hand-feeding was also employed. Before the mid-eighteenth century it appears to have been more common to send children out to rural parishes to wet-nurse, rather than to employ live-in nurses. This practice can be detected in the large numbers of burials of ‘nurse children’ in the burial registers of rural parishes near London (Clarke, 1987; Fildes, 1986, pp. 152–163, 1988b; Finlay, 1981, pp. 146–149). Paternal occupations recorded in the burial entries of nurse children in rural parishes indicated that the employment of rural wet-nurses was common to a wide range of social status groups from gentry and professionals to small-scale traders, although most frequent amongst wealthier families (Clark, 1987; see also Fildes, 1986, pp. 99–100).

Burials of private nurse children were very common in rural parish registers before 1750, but the practice seems to have declined precipitously in the mid-eighteenth century, at least in the parishes examined by Fildes (Fildes, 1988b). Fildes also argued from qualitative evidence that wet-nursing became less common in the eighteenth century as a consequence of a growing aversion to the practice. This aversion manifested initially in experiments with hand-feeding, as well as live-in wet-nursing, but developed in the second half of the eighteenth century into a new fashion for maternal breastfeeding amongst elite families (Fildes, 1986, pp. 106, 288–292). Fildes also argued that the increasing willingness of mothers to breastfeed, especially in the first few days of life, contributed to the marked fall in neonatal mortality in the population as a whole over the eighteenth century (Fildes, 1980, 1986, pp. 87–91).

Infant-feeding practices were a major determinant of infant survival in historical populations, and show remarkable variation both geographically and socioeconomically (Thorvaldsen, 2008). Therefore changes in the prevalence of breastfeeding had the potential to influence mortality trends and to widen or narrow socioeconomic and urban-rural differentials in infant survival. The evidence for strong socio-economic gradients in infant mortality before the mid-nineteenth century in north-western Europe is very mixed, in contrast to the almost ubiquitous gradients observed in twentieth century populations (Bengtsson, 2004; Bentsson & van Poppel, 2011; Oris, Derosas, & Breschi, 2004; Reid, 1997). Differences in the incidence and duration of breastfeeding by social class may have contributed to the muting of socioeconomic differentials in infant mortality in historical populations. Conversely, some of the observed differences in infant mortality by familial wealth may reflect the differential use of rural wet-nursing, and associated under-counting of infant burials (e.g. Bengtsson & van Poppel, 2011).

English demographic sources rarely provide systematic evidence of social status before 1813, but what very limited evidence exists suggests little survival advantage of wealth before the nineteenth century (Smith & Oeppen, 2006). With respect to London, London Quakers were relatively affluent compared with the London Bills population as a whole, and the similarity in infant mortality rates between the two groups suggested that wealth may have conferred little advantage in this period, at least in childhood (Landers, 1993, Chapter 5; Laxton & Williams, 1989). Levene’s estimates of mortality of infants abandoned at the London Foundling Hospital (1741–99) also suggested a decline in mortality amongst some of the most disadvantaged children in London, although her rates referred mainly to the experience of children at nurse outside London (Levene, 2005, 2007). Razzell and Spence (2007) reported similar levels and trends in infant mortality between ‘elite’ and non-elite families in several London sources between 1538 and 1850, but the numbers involved were small and the quality of the sources problematic. In this paper we used data representing (almost) the full social spectrum to quantify the extent and impact of changes in infant feeding and care in London in the period 1752–1812, and to determine to what extent these effects were shared across social classes in the metropolis. We first describe the sample and test the credibility of our status categories. We then present analyses of birth intervals and the seasonality of mortality in the sample because these provide indirect evidence of infant-feeding practices. Evidence of unobserved removal of children, probably for wet-nursing, made it necessary to apply adjustments to aggregated mortality rates to account for differences in the extent of missing children between status groups and over time.

## 2. Sources and methods

St. Martin in the Fields was a large Westminster parish of 25–30,000 inhabitants throughout the period 1750–1851. Its population grew as part of the westward expansion of affluent London suburbs in the seventeenth century, and its proximity to the royal court at Westminster attracted wealthy inhabitants throughout the eighteenth and early nineteenth centuries. However the sheer size of the parish meant that it provided most of the functions typical of a sizeable town. Its population included a wide range of occupational and socioeconomic groups in addition to elite families, and included an excess of unmarried women drawn to work as domestic servants (Boulton, 2000; Davenport, 2016, fn. 6). By 1801 its workhouse was the third largest in London (Parliamentary Papers, 1803–04).

The parish has exceptional records for demographic purposes. In addition to the Anglican parish baptism and burial registers, fee books have survived that detail payments for these services. The baptismal fee books recorded the fees paid for baptism, the street address and names of the parents, and the date of birth as well as baptism. The date of birth was key to measuring mortality by age, because parents often waited months before baptising children, and so baptism dates could not be treated as proxies for birth dates (Boulton & Davenport, 2015). From 1752 the sextons’ burial books recorded age at death, cause of death (including ‘stillborn’ burials usually omitted from burial registers) and street address of the deceased, as well as a breakdown of the fees paid. Moreover unlike the parish register, which was a record of Anglican burials in the parish, the sexton’s burial books also included details of corpses of residents that were exported for burial in other parishes. The information on address and exported burials revealed a lively market for burials in eighteenth-century London, and made it possible to quantify the traffic in corpses between parishes that could otherwise bias measures of mortality rates (Boulton, 2014).

The key problem with the use of parish registers to estimate mortality rates is to establish the denominator for the rates, that is the population at risk of death. This is particularly difficult in urban populations because of their extraordinary mobility. Towns were characterised by high rates of in- and out-migration, and also by very high levels of intra-urban residential mobility. Eighteenth-century London included over a 100 parishes, and individuals and families moved frequently across parish boundaries in a kind of Brownian motion (Boulton, 1987; Davenport, 2016). Furthermore, in London at least there was a market for burials that resulted in high volumes of extra-parochial burials (Boulton, 2014). In the light of these problems we used a ‘partial’ family reconstitution methodology to link infants born in the parish to their parents and siblings (Davenport, 2016). We then assumed that infants remained ‘in observation’ in the parish, and therefore at risk of dying and being recorded as buried in the parish, if their families went on baptising subsequent children in the parish. In practice however, we found that many families moved frequently and often spent spells dwelling outside the parish (and registering baptisms and burials elsewhere). Therefore we restricted our analyses to periods when families apparently resided at a single address. This did not overcome the problem of rural wet-nursing, and this is investigated later in the paper. The very high residential mobility of the population of St Martin in the Fields meant that most families in our sample were only in observation for a few years. We therefore report only mortality rates in the first two years of life, where record linkage was most reliable (Davenport, 2016, see also Appendix 1).

Information on social status or wealth is rare in English historical samples, and is usually biased towards the more affluent (such as those who left wills, paid taxes or voted), or paupers (documented in poor law sources). However in St. Martin’s the baptismal fee books recorded fees paid for all baptisms. Baptism was expensive and baptism fees showed substantial gradation in the period before 1795 (ranging from free to 2,652 pence or £11 and 1s) (Boulton & Davenport, 2015). The daily wage of a building labourer in London for most of the eighteenth century was 24 pence a day (when employed), making even the standard fee of 18 pence for public baptism a not inconsiderable expenditure (Schwarz, 1985). After 1794 baptism fees collapsed to a two-tier structure of pauper (free) and standard 18 pence fees, and analysis by wealth could not be extended beyond 1794.

For the period 1752–1794 reconstitution families were assigned to one of four wealth categories that reflected the structure of baptism fees and provided adequate differentiation without creating groups with too few members for analysis. The four groups were based on the distribution of baptism fees in the parish, which were heaped on pauper baptisms (no fee), public baptisms (18 pence), private baptisms (42 or 60 pence) and more expensive baptisms (in practice mainly 100+ pence). There were gaps in survival of the baptism fee books, when we were forced to rely on the baptism registers that did not record fee. These periods were short enough that most baptisms linked to reconstitution families in these periods belonged to families where the fee was known for at least one other baptism. Similarly, infants who died before baptism lacked fee information. In some cases families paid different fees for successive baptisms. Families were assigned to the wealth category of the modal baptism fee paid by the family (the lowest modal fee where there was more than one modal value). We chose to allocate families a single wealth status, rather than allowing status to vary, because nearly 20% of births lacked fee information, making more fluid measures less sensitive indicators than would otherwise be the case.

Our reconstitution sample contained fewer pauper baptisms than the sample as a whole, due in part to the exclusion of illegitimate births. Reconstituted families also included more high-cost baptisms than the sample as a whole, because wealthier families were less residentially mobile than poorer families and were therefore more likely to be included in the reconstitution and to remain in observation for longer (Table 1).^1^ Reconstitution families also included more births that lacked fee information than the sample as a whole, because they included infants who died before public baptism (Davenport, 2016). When families were allocated to social status categories using the method described then the proportions of births in families in the higher-status categories increased further, because more of the infants who died before baptism and were assigned ‘dummy’ baptisms were from wealthier families. The method had the virtue of distributing most of the large fraction of baptisms with no fee given into known status groups, and created four groups of sufficient size for analysis. There was a reduction over time in private baptisms in the parish which was reflected in the growth of the ‘standard baptism’ category at the expense of the ‘private baptism’ category of families (Boulton & Davenport, 2015). From 1795 it was only possible to distinguish pauper families (4% of the sample) from non-paupers (96%).

**Table 1.** Percentage distribution of baptisms by fee and family social status, 1752–1794.

Baptism fee	All baptisms in parish	Reconstitution family births	Reconstitution family social status
0 (pauper)	11.9	5.7	9.6
1–39 pence	48.5	41.4	38.5
40–99 pence	23.2	28.6	32.1
100+ pence	4.3	6.0	18.1
not given	12.0	18.3	1.7
N	31,680	13,430	13,430

*Notes*: infants who died without registered baptisms (N = 688) are included in columns 3 and 4.

*Sources*: City of Westminster Archives Centre (COWAC) St Martin in the Fields baptism registers and christening and marriage fee books, Accession 419/210–227.

A key question is the extent to which baptism fees provided a useful proxy for the actual wealth of the reconstitution families. We used two other sources of information on wealth to assess this. The first was the Westminster rate books, which listed rate-payers and the estimated rental value of their dwellings (the basis of the assessment for poor rates). The only rate book available in machine-readable form for the period 1752–94 was that for 1784 (Westminster Historical Database, UK Data Service SN 3908). We matched male ratepayers in 1784 to fathers named in baptisms in the years 1783–85 using name and street address.^2^ We were able to link 33.7% of baptisms in reconstitution families to an entry in the rate book, and the proportion linked rose with baptism fee (Table 2). Only two pauper baptisms could be linked to a rate book entry, presumably because most families deemed pauper were too poor to live in properties of sufficient rental value to be rateable (although the higher mobility of poorer families may also have reduced linkage rates for groups 0 and 1). Assessed rental values were also positively and significantly related to baptism fee, although there was considerable overlap particularly between the two higher-status groups (Table 2).

**Table 2.** Assessed rateable values by family baptism fee category, 1783–85.

Family baptism fee group	Mean assessed value (£)	95% confidence intervals	Number of linked baptisms	Total baptisms 1783–85	% linked
0 (pauper)	n/a	n/a	2	95	2.11
1 (1–39 d)	19.24	17.13–21.35	180	672	26.79
2 (40–99 d)	29.79	24.53–35.04	122	204	59.80
3 (100+ d)	38.68	33.66–43.70	63	119	52.94
Total			367	1,090	33.67

*Sources*: as for Table 1; Westminster Historical Database, UK Data Service SN 3908.

The second source of information on wealth was the fee paid for burial. While baptism was not cheap burial was considerably more expensive, with an elaborate schedule of fees according to where and when the burial took place, and with what ceremony (Boulton, 2014). As with property rates there was both a clear gradient in fees with social status, and some overlap in burial fees between social status groups (Table 3).

**Table 3.** Cumulative percentage distribution of child burial fees in reconstitution families.

	Family social status from baptism fees				
burial cost (pence)	0 (pauper)	1 (1–39 d)	2 (40–99 d)	3 (100+ d)	N (burials)
0–49	83.7	12.2	7.9	4.0	141
50–99	85.4	69.7	35.8	24.2	2,251
100–199	98.0	92.1	76.0	36.9	1,205
200+	100	100	100	100	692
N	398	2,623	1,330	252	4,603

*Sources*: see Table 1; St Martin in the Fields sextons books and burial books (COWAC, MS 419/233–44; 419/265–269; f2465, F2467, F2469).

On the basis of comparison with property rates and with burial fees it therefore appeared that the four groups defined by baptism fees represented a definite gradient in wealth.

## 3. Results and discussion

### 3.1. Birth intervals and infant-feeding practices

We start our analysis with an investigation of the length of time between births in reconstitution families, because breastfeeding habits exerted a major influence on birth spacing. Birth intervals also provide a check on data quality, especially regarding the registration of infant deaths, something we consider in the next section.

The interval between successive births consists of the gestation time (9 months), waiting time to conception and effects of miscarriages (usually estimated at 2–3 months), and the variable effects of artificial contraceptives, abortion, breastfeeding, sexual abstinence, and fecundity (Bongaarts & Potter, 1983). In the English historical context it is generally assumed for the period c. 1550–1870 that the main determinant of birth intervals, above the biological minimum of c. 12 months, was breastfeeding. When of sufficient intensity and duration, breastfeeding suppresses ovulation and so prevents conception, a phenomenon termed ‘lactational infecundability’. This can exert profound effects on fertility, and was associated in the English case with average birth intervals of around two and half years (Wrigley et al., 1997, p. 447). In addition to breastfeeding there is also anecdotal evidence in the early modern English population for other practices that could have acted to reduce fertility and therefore to increase the intervals between births, including abortion, artificial contraception, and abstinence. In his sustained argument for pre-transitional fertility control in the English population McLaren succeeded admirably in demonstrating the demand for and variety of contraceptive techniques and abortion in early modern England. However, in terms of the *effectiveness* of these strategies in achieving birth spacing, he privileged ‘extended nursing, possibly abetted by continence or contraception’ (McLaren, 1990, p. 142). In what follows we focus on breastfeeding as the main determinant of variations in birth intervals in the English population in this period, but also consider alternative possibilities.

**Figure 2.**
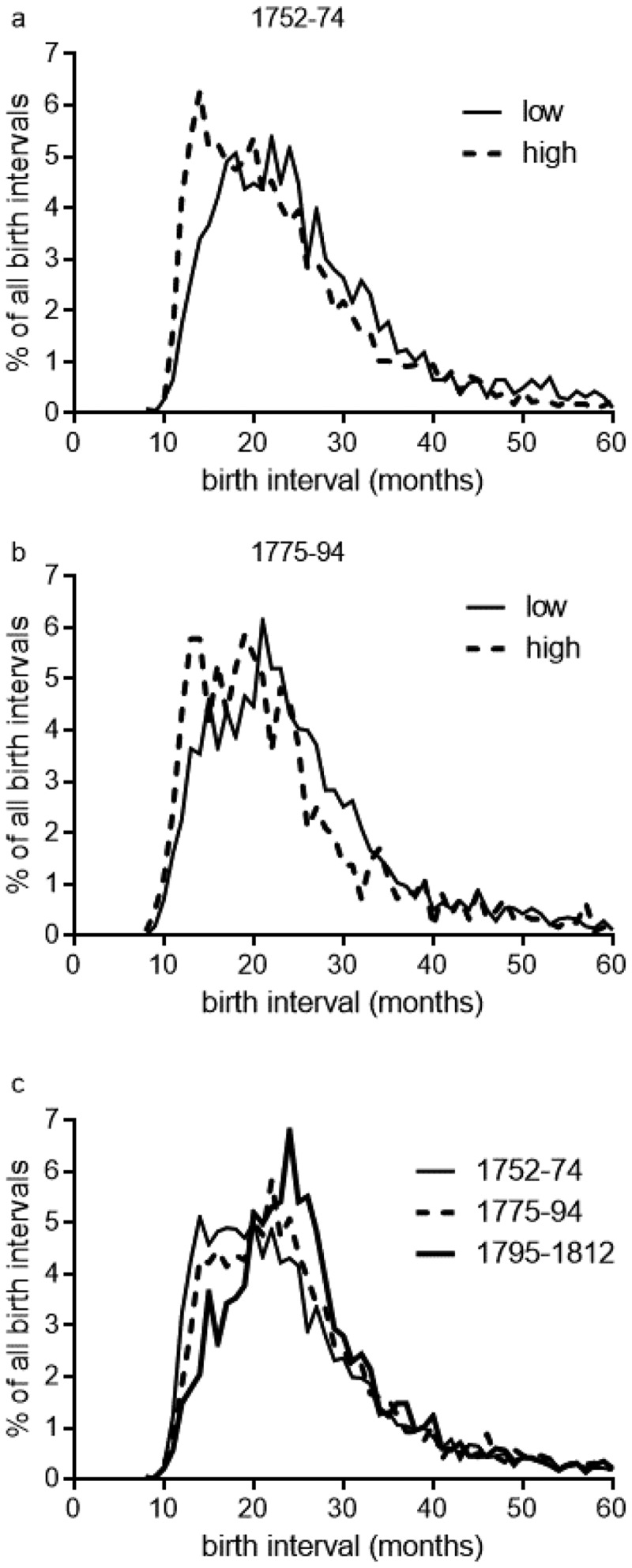
Percentage distribution of birth intervals by familial wealth. *Notes*: ‘Low status’ families included groups 0 (paupers) and 1, ‘high-status’ families comprised groups 2 and 3 (highest cost baptisms). Numbers of birth intervals are given in Table 3.Source: St. Martin in the Fields family reconstitution.

**Figure 3.**
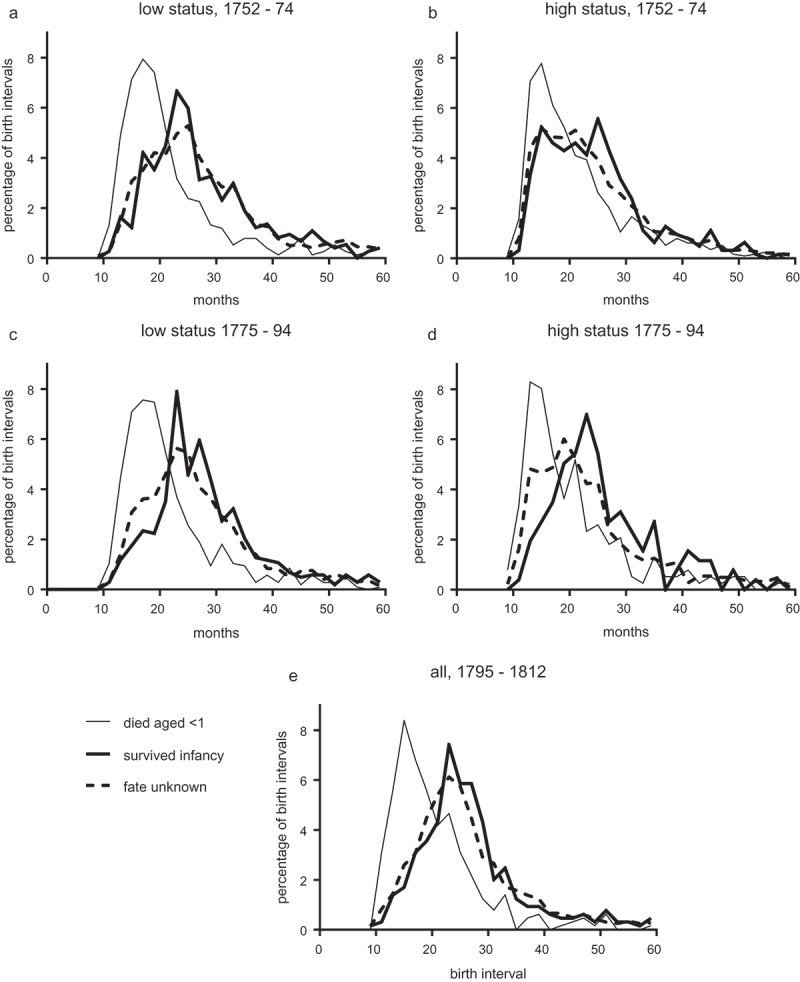
Percentage distribution of birth intervals by familial wealth and fate of the infant whose birth opened the interval. *Notes*: ‘Low status’ families included groups 0 (paupers) and 1, ‘high-status’ families comprised groups 2 and 3 (highest cost baptisms). Numbers of birth intervals are given inTable 5.Source: St. Martin in the Fields family reconstitution.

While birth intervals are easily measured in family reconstitution studies, they are problematic to analyse because birth interval distributions tend to be highly right-skewed, with a long tail of very long durations that can’t be analysed using standard parametric techniques such as comparison of means (Figures 2, 3).^3^ In the analyses that follow we therefore present plots of the full monthly distributions of birth intervals as well as means and other summary measures (as presented in Tables 4 and 5), because the latter are insufficient to capture the characteristics of the distributions. To compare the distributions of birth intervals between groups or periods we used a non-parametric test, the Kolmogorov-Smirnov test that measured whether the distributions differed significantly. Unfortunately the smaller status groups, the wealthiest and paupers, were too small for meaningful analysis in some cases, and so for some analyses two groups were created of ‘high-status’ (private and higher baptismal fee-paying) and ‘low status’ (pauper and standard fee-paying) families.

**Table 4.** Birth intervals by social status.

	Social status				
	0 (pauper)	1	2	3 (highest)	all
1752–74					
Mean	30.3	26.8	24.4	24.6	26
Median	26	24	21	20	22
N	240	1,674	2,434	391	4,739
1775–94					
Mean	28.3	27.0	26.1	25.3	26.7
Median	26	24	22	21	23
N	272	2,647	748	542	4,209
1795–1812	pauper	non-pauper			all
Mean	25.2		27.5		27.4
Median	23		25		25
N	213		2,692		2,905

*Source*: St. Martin in the Fields family reconstitution.

**Table 5.** Birth intervals by family status and fate of first child in interval.

	Low status			High status		
	Died < 1 year	Survived infancy	Fate unknown	Died < 1 year	Survived infancy	Fate unknown
1752–74						
mean (months)	22.2	28.1	28.7	22.2	25.0	25.0
median	18	25	25	18	23	22
P same as low status group				0.13	0.00	0.00
N birth intervals	384	378	1,152	584	320	1,921
1775–94						
mean (months)	22.4	29.3	27.9**	22.1	27.6	26.2**
median	18	26	25	18	24	22
P same as previous period	0.54	0.00	0.42	0.99	0.01	0.33
P same as low status group				0.14	0.07	0.00
N birth intervals	539	533	1,847	196	132	962
1795–1812 (status groups combined)						
mean (months)	21.9	28.7	28.0			
median	19	26	25			
P same as previous period	0.70	0.55	0.00			
N birth intervals	327	334	2,244			

*Notes*: P values derived from Kolmogorov-Smirnov tests for equality of distributions of birth intervals. Asterixes indicate that the ‘fate unknown’ group differed from the ‘survived infancy’ group with P < 0.05 (*) or P < 0.01 (**).

*Source*: St. Martin in the Fields family reconstitution.

Table 4 and Figure 2 present information on birth intervals by familial wealth, for all birth intervals (regardless of the infant’s fate). In the third quarter of the eighteenth-century birth intervals were inversely related to wealth. The mean birth interval in pauper families was 30 months, or two and a half years between births, compared with just 25 months in the wealthiest families (Table 4, first row). Figure 2(a) shows the percentage distribution of birth intervals by familial wealth, 1752–74. Birth intervals were clearly shorter amongst wealthier families, with a substantial peak of very short birth intervals close to the biological minimum of 9–12 months. There was a lengthening of birth intervals amongst all wealth categories except paupers in the period 1775–94 (Table 4, Figure 2(b)). In the period 1795–1812, when we could only distinguish paupers and non-paupers, the distribution for the reconstitution population as a whole had shifted decisively to much longer birth intervals, with a mean birth interval of 27 months (Figure 2(c), Table 4).

Table 4 and Figure 2 describe all birth intervals in the sample, and therefore the shifting patterns they report include the effects of any changes in both infant mortality and in breastfeeding habits. In the English population in the eighteenth century average birth intervals (where the first child survived) were around 30–33 months, consistent with extended breast-feeding of perhaps 14 to 18 months’ duration (Wilson, 1986; Wrigley et al., 1997, pp. 430–449). However if an infant died before weaning then this caused a cessation of breastfeeding and an early resumption of fecundity, resulting in a shorter interval to the next birth (Wrigley et al., 1997, pp. 101–106). The effect of early death on breastfeeding made it possible to tease out the likely contribution of breastfeeding to birth intervals, by comparing the interval to the next birth in cases where the first infant survived, and where it died in infancy. In cases where the first infant was known to have survived infancy, the interval to the next birth should reflect how long the mother chose or was able to breastfeed that infant. The third remaining possibility, that the fate of the infant was unknown, is explored later.

Figure 3 shows the distributions of birth intervals associated with the three possible fates of infants whose birth opened the interval. Results are reported in only two aggregated wealth categories to maintain valid sample sizes. In the case of infants who died early (in the first year of life) the interval to the next birth was short, because the infant’s death curtailed any breastfeeding (thin black lines, Figure 3; Table 5). There was a substantial peak close to the biological minimum of roughly 12 months, and mean birth intervals were 22 months. In contrast, for infants known to have survived the first year of life average intervals to the next birth were much longer, with mean values of 25–29 months, suggesting that maternal breastfeeding was practiced to some extent and delayed conception where the breastfed infant survived long enough (heavy black lines, Figure 3; Table 5). However, differences in birth interval distributions between the two status groups were striking. In the period 1752–74, in the case where infants were known to have survived infancy, birth intervals amongst lower-status families were distributed with a single peak at 23 months (Figure 3(a)), a pattern suggesting slightly shorter average birth intervals compared with the national Cambridge Group reconstitution sample (compare the distributions in Wrigley et al., 1997, Figure 4.2, p. 104). In contrast the survival of infants in higher-status families was associated with a wide, possibly bimodal distribution of birth intervals suggesting substantial heterogeneity in behaviours (heavy black lines, Figure 3(b)). Some proportion of higher-status infants who survived infancy were apparently breastfed by their mothers for only a very short period or not at all, resulting in a cluster of birth intervals as short as was the case where the infant died within the first year of life.

These patterns corroborate Fildes’s claims that high-status women rarely breastfed their infants in early modern England. Our ‘high-status’ group represented families in the upper half of the wealth distribution in 1752–74, and appears to have included a mix of breastfeeding practices, with a substantial proportion apparently breastfeeding very little or not at all.

We could also use these data to examine Fildes’s claim for a rising preference for maternal wet-nursing, by following trends in birth intervals between 1752 and 1812. Remarkably, in the second period, 1775–94, in cases where infants were known to have survived the first year of life, birth intervals amongst wealthier families shifted towards a pattern resembling that of lower-status families, with a single peak at 23–24 months (Figure 3(c,d) Table 5).^4^ This trend to longer birth intervals was sustained into the early nineteenth century, when the distribution of birth intervals amongst surviving infants in the sample as a whole was almost identical to that of poorer families in the earlier periods (Figure 2(c); Table 4).

The simplest explanation for the convergence in birth intervals between wealthy and poor families is a substantial increase in the prevalence and/or duration of maternal breastfeeding amongst wealthier families over the second half of the eighteenth century. This is consistent with qualitative evidence regarding the adoption of maternal breastfeeding in elite families. An alternative explanation, that high-status women adopted other means apart from breastfeeding to space or prevent births, cannot be assessed with these data. However the evidence that birth intervals of wealthier families converged to the norm of poorer families would suggest that any deliberate contraceptive behaviour on the part of wealthier families did not result in longer birth intervals than those achieved by pre-existing breastfeeding norms.^5^

### 3.2. Seasonality of infant mortality

A second indirect way of investigating breastfeeding habits is via analyses of the seasonality of mortality by age. Breastfeeding provides optimal nutrition for infants, and also provides generalised protection against both gastrointestinal and respiratory infections. However upon weaning infants are exposed to multiple risks in the form of inadequate nutrition and infection, especially from contaminated foods. The latter risk is highest in summer, because higher temperatures accelerate growth rates of food-borne pathogens, and also of flies and other vectors of disease. Therefore a summer peak in infant mortality is often taken as evidence of high rates of gastrointestinal infection, and therefore of non-universal maternal breastfeeding. However it is important to note two caveats. First, other diseases were also associated with late summer peaks, including smallpox. Second, gastrointestinal infections did not necessarily imply an early cessation of breastfeeding. Fildes cited a number of seventeenth and eighteenth-century advice manuals that recommended the avoidance of colostrum, the nutrient- and antibody-rich milk produced in the first few days after delivery. Instead newborn infants were recommended to be fed alternative substances as ‘purges’, that often included almond oil and sugar or honey (Fildes, 1980). In this case infants whose mothers intended to breastfeed could nonetheless have been exposed to highly contaminated foods in the first few days of life. This could have resulted in summer peaks in neonatal mortality despite widespread breastfeeding of infants with post-colostrum milk. Fildes attached great importance to the practice of avoiding colostrum, claiming that the decline of this practice in the eighteenth century made a major contribution to improvements in neonatal mortality in this period (Fildes, 1980, 1986, pp. 88–89).

The seasonality of mortality (for neonates) and deaths (for ages 1 month and above) is presented in Table 6. It was necessary to group deaths by quarters, and to combine all social groups to maintain adequate sample sizes. Neonatal mortality displayed a prominent summer peak throughout the period 1752–1812. This was in strong contrast to the relatively aseasonal pattern of endogenous mortality in the wider English population, and points to a major contribution of infectious diseases with greatest effect in the summer months. As was the case amongst London Quakers, this summer peak in neonatal mortality persisted in St. Martin in the Fields despite lengthening birth intervals and significant falls in neonatal mortality (documented below). In common with London Quakers, there was also a summer peak amongst slightly older infants, aged 1–5 months, but no summer peak at older ages. This summer peak in mortality of the youngest post-neonates disappeared after c.1795. At ages six months to two years mortality was concentrated in winter and early spring. Since this was the age group most affected by smallpox, deaths from which peaked in October, the difference in patterns between in older children and young infants indicated that smallpox was not the cause of the summer peak amongst infants.

**Table 6.** Seasonality of neonatal mortality and post-neonatal deaths, relative to annual average.

	Jan–Mar	Apr–Jun	July–Sep	Oct–Dec	N (burials)
0–28 days					
1752–74	0.92	0.84	**1.23**	1.02	287
1775–94	0.89	0.94	**1.25**	0.92	200
1795–1812	0.52	0.92	**1.47**	1.09	93
1–5 months					
1752–74	0.90	0.97	**1.14**	0.98	511
1775–94	0.96	0.94	**1.19**	0.91	411
1795–1812	**1.29**	0.91	0.96	0.84	198
6–11 months					
1752–74	**1.16**	0.90	0.86	1.08	423
1775–94	**1.25**	0.76	0.95	1.04	372
1795–1812	1.14	0.85	0.77	**1.25**	218
12–24 months					
1752–74	**1.42**	0.99	0.66	0.93	535
1775–94	1.09	**1.14**	0.79	0.97	561
1795–1812	1.10	**1.23**	0.69	0.98	340

*Notes*: Neonatal mortality rates were calculated per quarter and corrected for differing lengths of month. For older age groups the seasonal distribution of deaths is reported, corrected for differing lengths of months (but not for seasonality of births, which was very muted).

*Source*: St. Martin in the Fields family reconstitution

These patterns in the seasonality of infant and child mortality are not straightforward to interpret with respect to breastfeeding, but are consistent with very early weaning or food supplementation. The summer peak of mortality at ages 1–5 months suggested some hand-feeding in this age range. The disappearance of this peak after c.1795 coincided with substantial reductions in the numbers of very short birth intervals amongst high-status families, and is consistent with an increased duration of breastfeeding in this period (Figure 3). Given this, the persistence of an exclusively neonatal summer peak is suggestive of the use of non-breast milk ‘purges’ close to birth, and the persistence of such practices throughout the period. The absence of a summer mortality peak at ages 6–23 months is also anomalous. In the non-metropolitan population there was a pronounced summer excess mortality associated with the estimated age at weaning, of c. 18 months (Wrigley et al., 1997, pp. 340–347). It seems plausible, however, that the heterogeneity of childcare practices in St. Martin’s, evident for instance in the persistence of wet-nursing documented in the next section, led to a wider range of weaning ages and prevented a clear age-season relationship from emerging amongst older infants and young children.

### 3.3. Missing infants and rural wet-nursing

The existing literature on infant feeding suggested that many infants in our sample may have been removed to rural parishes for wet-nursing. We couldn’t observe this practice directly, but if present it would undermine a key assumption of family reconstitution, that so long as families continued to baptise infants at the same street address then the children could be assumed to be resident in the parish. Rural wet-nursing presented a problem for our purposes because many wet-nursed infants died and were buried in their nurse’s parish, leaving no record of their death in the parish of their parents. This would cause us to underestimate mortality amongst families who used extra-parochial wet-nurses. In her study of infant mortality in London between 1550 and 1750 Newton was able to document this problem by comparing census-type data derived from the imposition of the Marriage Duty Assessment Act in 1695 with family reconstitution data for six London parishes. She established that children aged under three years from wealthy families were particularly likely to be excluded from the census, suggesting that they were living elsewhere even though their families remained in London (Newton, 2011). This paucity of young children in the wealthier parishes was associated with markedly shorter birth intervals in these parishes, consistent with the practice of wet-nursing and lack of maternal breastfeeding, an association also noted by Finlay (1981, pp. 133–150).

Infants raised in St. Martin’s could also escape observation after death, if the corpse was exported for burial in another parish, and the death was not recorded in the parish burial books.

To detect the extent to which infants were missing from observation we used birth interval analyses. This method relies on the third category of infants presented in Figure 2, those for whom no further record could be found. In our study we followed infants to age five, and attempted to link them to their own burials. This produced two sets of infants with known fate: those who died in infancy, and those who were known to have survived infancy, because they died later and could be linked to a burial record. However the majority of infants could not be linked to a burial at all, and so their fate was unknown. This group included infants who survived beyond the age of five and remained in the parish, but could also include infants who died and whose burial was unrecorded, or who were removed from the parish in infancy while their family remained in observation. Critically, in the case where the infant survived beyond age five and remained in the parish, then we would expect the distribution of birth intervals to be very similar to that of infants who were known to have survived infancy and remained in the parish. However if the infant died or left the parish in infancy, then we would expect the interval to the next birth to be shorter, because of the curtailment of breastfeeding, caused by the death or removal of the infant. The method is detailed by Wrigley et al. (1997, pp. 100–106).

The distributions of birth intervals following the birth of an infant of unknown fate were skewed towards shorter birth intervals compared with those for infants known to have survived the first year of life (dashed versus heavy solid lines in Figure 2(b–e); Table 5). This suggested that breastfeeding was more likely to have been cut short, or never initiated, amongst infants of unknown compared with known fate. The problem was more marked in the wealthier group, but was most pronounced, in both wealthy and poorer families, in the period 1775–94, when birth intervals were significantly shorter in the unknown fate group than amongst infants known to have survived infancy (Table 5, asterixed values).

We could calculate the number of infants associated with anomalously short birth intervals by regressing the distribution of birth intervals where the fate of the first infant was unknown as a function of the distributions of birth intervals where the first infant died early or survived infancy (Wrigley et al., 1997, p. 106).^6^ The numbers of missing infants calculated in this way for each period are shown in Table 7, and represent 22–23% of all births in the periods 1752–94, but only 16% of all births after 1794. The extent of anomalous birth intervals was highest amongst higher social status groups, with c.40% of infants apparently escaping observation early in the wealthiest group in the period 1752–94.

**Table 7.** ‘Missing’ infants predicted from birth interval analyses.

	Social status			
	0 (pauper)	1	2	3 (highest)
1752–74				
Births	347	1294	2224	881
Missing from observation	70	131	510	400
% missing from observation	20.2	10.1	22.9	45.4
1775–94				
Births	455	2054	710	898
Missing from observation	51	368	141	343
% missing from observation	11.2	17.9	19.9	38.2
1795–1812		(non-pauper groups combined)		
Births	197	2630 424		
Missing from observation	32			
% missing from observation		16.2		

*Notes*: The percentages of infants deemed ‘missing’ from the total of infants of unknown fate were estimated from birth interval analyses performed on grouped data for high (groups 2–3) and low (groups 0–1) status groups and were used to estimate the percentage ‘missing’ in individual status groups (see footnote 6). For the period 1795–1812 percentages missing were estimated for all groups combined (paupers and non-paupers).

*Source*: St. Martin in the Fields family reconstitution.

The very high proportions of infants apparently missing from observation could be explained by either unobserved infant deaths in the parish (because the infants were buried elsewhere), or by the removal of infants for wet-nursing. We concluded that the latter explanation was more likely, because the social gradient in missing infants matched more closely the social gradient in wet-nursing than that of ‘clandestine’ burial exports, where cost was a major motive (Boulton, 2014; Davenport, 2016). If this is correct, then the patterns described above would suggest that almost a third of infants from wealthier families were sent for rural wet-nursing in the mid-eighteenth century, and that this proportion declined slightly amongst higher-status groups in the last quarter of the century while gaining in popularity amongst lower-status families.^7^ However by the first decade of the nineteenth century the practice was in steep decline. The apparent demise of rural wet-nursing would account for the decisive shift to longer birth intervals evident in the sample as a whole in the early nineteenth century (Figure 1(c)), and would corroborate the shift to maternal breastfeeding over the period.

The evidence that significant numbers of infants escaped observation in the first two years of life, despite the continued presence of their families within the parish, meant that mortality rates would be under-estimated without adjustment for these missing infants. Tests described in Davenport (2016) indicated that registration of *early* neonatal deaths was almost complete, once the practice of burying unbaptised infants as ‘stillborn’ was accounted for. The classic test for under-registration of burials, the Bourgeois-Pichat or biometric test, also indicated no shortfall of deaths of newborn infants (Appendix 2). Rather it appeared that specific processes affecting the registration of burials of *older* infants resulted in significant omission of burials at these ages. This age-specific pattern of missing burials was consistent with the tendency to delay sending children out to nurse until after baptism (Fildes, 1988a, pp. 81–83).^8^ It was, however, also consistent with the age pattern of exported burials, which were very rare in the first week after delivery, but which rose to 10–14 percent of all burials for post-neonatal children (Davenport, 2016, Table 9). The methodology for calculating and adjusting mortality rates is described in Appendix 3.

### 3.4. Mortality in the first two years of life, by familial wealth and period

**Figure 4.**
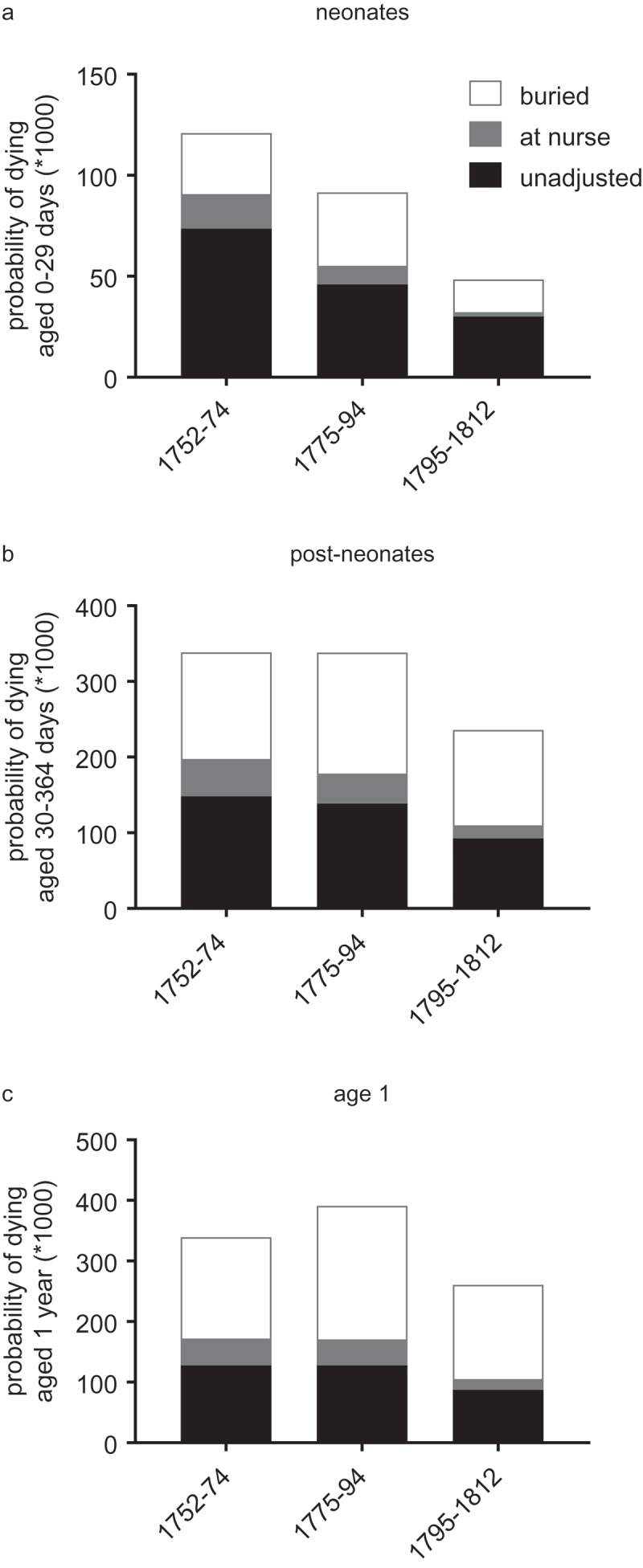
Age-specific mortality rates by period, assuming no missing infants (‘unadjusted’), missing infants sent to nurse, or missing infants buried without record. *Notes*: Adjustments to mortality rates are detailed in Appendix 3. *Sources*: St. Martin in the Fields reconstitution.

Figure 4 reports probabilities of dying by age for the reconstitution sample as a whole, adjusted under the assumptions that (1) recording of infant burial was complete (‘unadjusted’), (2) the proportion of infants identified as missing were all sent to nurse (and so were excluded from analysis), or (3) missing infants all died in infancy. The levels of mortality were clearly higher in both adjustment scenarios, and highest where all missing infants are assumed to have died. Indeed in the case where missing infants were all presumed dead then death rates were improbably high, especially in the second year of life, lending some credence to the hypothesis that many infants were sent out of the parish alive to nurse rather than exported in coffins. It is likely that both practices, of rural wet-nursing and uncertificated burial export, occurred, and may have varied in their levels over time and between social status groups, but we had no grounds for any further adjustments to mortality rates on this basis. Under the most plausible assumption that missing infants were sent to nurse then over a quarter of live-born infants died in the first year of life in the period 1752–74 (270 deaths/1,000 births) but only 139/1,000 by 1795–1812. These rates were somewhat lower than those reported for London Quakers, but reassuringly higher than the national sample.^9^

Importantly, the chronology of improvements in mortality was fairly consistent at each age regardless of the adjustment procedure. Neonatal mortality improved markedly across the period, in common with London Quakers and the national Cambridge Group sample (Figure 4(a)). In the sample as a whole mortality improved little or not at all for older infants and one year olds in St. Martin in the Fields until the first decade of the nineteenth century (Figure 4(b,c)). This was in contrast to London Quakers, where there were significant improvements at all ages under 10 in the last quarter of the eighteenth century (Landers, 1993, p. 137). However the scale and chronology of mortality improvements differed by familial wealth in St. Martin’s, and wealthier families resembled the London Quakers more closely. Below we consider wealth-specific differences in mortality by age group, and focus on mortality rates adjusted for nursing. When missing infants were assumed dead then all advantages to wealth disappeared, and the wealthiest group especially experienced extremely high mortality, both absolutely and with respect to other groups.

**Figure 5.**
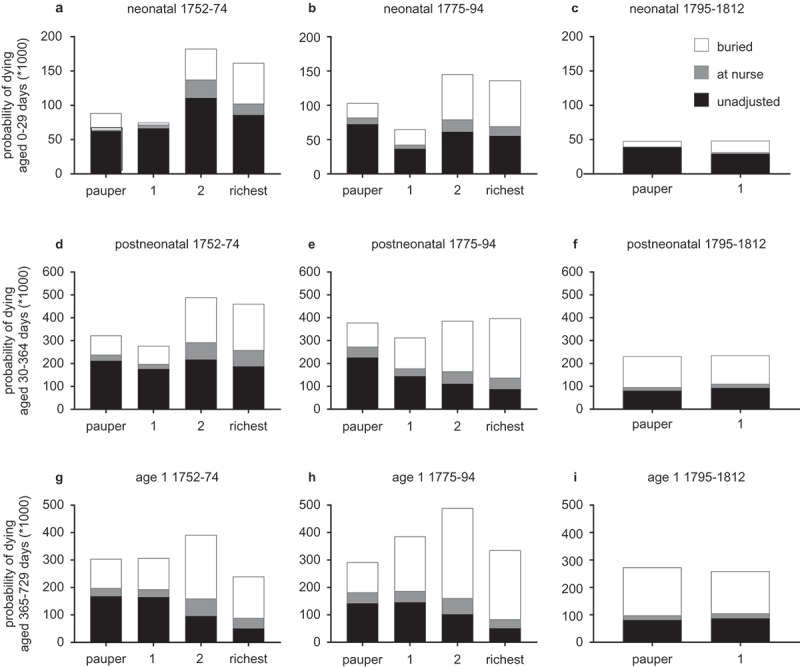
Age-specific mortality rates by familial wealth. *Notes*: Adjustments to mortality rates are detailed in Appendix 3.*Sources*: St. Martin in the Fields reconstitution.

Neonatal mortality is generally dominated by influences arising *in utero* (resulting in prematurity, low-birth weight or other problems) or during birth, when complications at delivery can result in injury. However where infant-feeding practices are sub-optimal then very young infants may also be at risk of infection or inadequate nutrition. In St Martin’s neonatal mortality was highest amongst infants of wealthier families in the period 1752–74 but improved markedly in the last quarter of the eighteenth century, in parallel with lengthening birth intervals in these families (Figure 5(a–c)). These data therefore suggested that infants of wealthier families may have been disadvantaged by a lack of maternal breast milk before the late eighteenth century, or by the consequences of short birth intervals for the health of their mothers.

However changes in breastfeeding habits amongst wealthier families could not account for all of the improvement in neonatal mortality in our sample, because neonatal survival also improved markedly amongst poorer families, where birth intervals suggested that long maternal breastfeeding was already the norm. By the early nineteenth century neonatal mortality had fallen very substantially amongst both paupers and non-pauper families, indicating improvements of very wide social effect (Figure 5(c)). These falls are consistent with the convergence in neonatal mortality rates between rural and urban populations and between high and low mortality environments over the same period evident in the Cambridge Group reconstitution sample and in the earliest returns of the Registrar-General in the 1840s (Smith & Oeppen, 2006; Wrigley et al., 1997, pp. 224–240, 279). Thus it seems likely that the particular mortality disadvantage of neonates of wealthy families in the third quarter of the eighteenth century was a function of high fertility and poor infant-feeding practices, but also that other more ubiquitous factors acted to drive down neonatal mortality in the population as a whole. Therefore broader changes, perhaps in maternal and foetal health, are also implicated (Smith & Oeppen, 2006; Woods, 2009; Wrigley et al., 1997, pp. 316–319, 1998).

Infants of wealthy families were also disadvantaged in later infancy (ages 1–11 months) in the period 1752–74, relative to poorer families (Figure 5(d)). As with neonatal mortality, this post-neonatal disadvantage disappeared in the last quarter of the eighteenth century, and a clear social gradient in survival emerged in this age group (adjusted for wet-nursing, Figure 5(e)). Mortality in this age range was dominated by infectious diseases, and gastrointestinal diseases could predominate where infants were not exclusively breastfed. As with neonatal mortality, a plausible explanation for the wealth-specific improvement in this age group in the period 1775–94 was the adoption of maternal breastfeeding suggested by the lengthening of birth intervals in wealthier families in this period (and the disappearance of a summer mortality peak at ages 1–5 months). The emergence of a positive social gradient in survival would then suggest that underlying advantages of wealth became evident once the disadvantages of elite feeding practices waned. However, by the first decade of the nineteenth century mortality had fallen very markedly in this age range and for paupers as well as the rest of the sample (Figure 5(f)). The equivalence of post-neonatal mortality rates between paupers and non-paupers in the period 1795–1812 suggested that there was again a marked narrowing or disappearance of any social gradient in mortality in this period. Therefore our evidence suggests that infants of wealthier families enjoyed a transient survival advantage in the late eighteenth century, but also that this advantage diminished or disappeared with some widespread improvement in post-neonatal survival at the turn of the nineteenth century that was relatively socially unselective.

In the second year of life infants of wealthier families were at lower risk throughout the period 1752–94, with infants from the wealthiest decile experiencing less than half the mortality rate of infants in the lower half of the wealth distribution (rates adjusted for nursing, Figure 5(g,h)). However as for post-neonates, this advantage of wealth apparently narrowed or disappeared again in the period 1795–1812.

A possible explanation for these phenomena, of a widening and subsequent narrowing of survival differentials by familial wealth in the period 1752–1812, is changes in the availability of prophylactics against smallpox. Smallpox was the leading cause of child mortality in eighteenth-century cities (Davenport, Boulton, & Schwarz, 2016; Davenport, Satchell, & Shaw-Taylor, 2018; Landers, 1993, Mercer, 2014), and Landers demonstrated that the large improvements in post-neonatal and child mortality of London Quakers in the second half of the eighteenth century were driven mainly by a specific reduction in smallpox mortality (although smallpox could not account fully for the fall in mortality in post-neonatal infants). Two forms of immunisation against smallpox became available in England in the course of the eighteenth century. The first was inoculation, which involved deliberate infection with a small dose of smallpox to induce a mild case of the disease that conferred lifelong immunity. Inoculation became popular in southern England from the 1760s, but apparently remained unpopular in cities and in northern Britain (Brunton, 1990; Razzell, 2003). While there is no evidence that inoculation was practiced on a large scale in London, it may have been used by some families, especially those who could afford the often expensive fee. The falls in smallpox mortality amongst London Quakers were initiated *before* the advent of vaccination, and suggest that Quaker families adopted inoculation in the late eighteenth century (Landers, 1993).

The safer technique of vaccination with cowpox was first publicised by Jenner in 1797, and enjoyed extraordinarily rapid success. Vaccination produced very rapid falls in smallpox mortality after 1800 in London and other large towns: in London smallpox fell as a cause of death from 9.4% of burials in the London Bills in the period 1775–1800 to 6.6% by 1801–12: in Manchester smallpox burials fell from 20.3% of all burials to 10.1% over the same period (Davenport et al., 2016). In the St. Martin’s sample as a whole, falls in mortality amongst post-neonates and one year olds were associated with specific falls in smallpox mortality after 1800 (Davenport et al., 2016). Unfortunately our sample was too small to permit analysis of causes of death by wealth. However it is plausible that the survival advantage at ages 30 days to 2 years amongst children of wealthier families in St. Martin’s before the advent of vaccination reflected the early adoption of inoculation against smallpox in these families. Amongst post-neonates this advantage may have been masked before the last quarter of the eighteenth century by the countervailing and baneful effects of low rates of maternal breastfeeding by more affluent mothers. However this advantage was greatly reduced or eliminated by the widespread and relatively egalitarian use of vaccination after c.1800.

## 4. Conclusions

This study is the first to report infant mortality by familial wealth across the social spectrum in Europe’s largest city in the first stages of the demographic transition. Although it was not possible to detect breastfeeding habits directly, our evidence suggested marked differences in infant feeding practices by wealth in the mid-eighteenth century, including artificial feeding and rural wet-nursing of infants especially in wealthier families. There also appears to have been a convergence in breastfeeding habits, to those of the poor, in the last quarter of the eighteenth century. This corroborates documentary evidence of a decline in artificial feeding in the least quarter of the eighteenth century. Our evidence is also consistent with a decline in the practice of rural wet-nursing of London infants over the same period, although our data suggest that the decline was not as abrupt as indicated by previous geographically constrained analyses of rural burial registers.

The reasons for the initial difference and subsequent large shift in the breastfeeding behaviour of high-status London women remain obscure. A number of societies have observed a taboo against sexual intercourse while breastfeeding, with very different behavioural consequences. In some populations this taboo was associated with very protracted sexual abstinence while breastfeeding, of up to several years’ duration (e.g. van de Walle & van de Walle, 1993). In contrast, Roman Catholic doctrine advised instead that women used wet-nursing ‘to provide for the frailty of her husband by paying the conjugal due’ (Fildes, 1986, p. 105). However, Fildes argued that Protestant societies entertained no such taboo, and that this probably contributed to relatively long maternal breastfeeding amongst the majority of the English population (Fildes, 1986, p. 105; Harley, 1995). Alternatively, the perceived difficulties of combining breastfeeding with social or economic functions may have been played a role in the low prevalence of breastfeeding specifically amongst higher status and urban English women (Fildes, 1988a, pp. 83–84). Where women were expected to accompany their husbands in London society, or could not combine breastfeeding with work, then wet-nursing provided a solution. Aristocratic families apparently preferred live-in wet-nurses, but most families who chose wet-nursing opted for rural wet nurses, probably for reasons of the cost and lack of domestic space (Fildes, 1988a, pp. 229–230; Marshall, 1988).

Another explanation sometimes given for rural wet-nursing was the widespread perception of the dangers of the dangers of urban disease environments, a view given official sanction in England by Hanway’s Act (1767), a bill requiring that pauper children in the care of London parishes must be send at least three miles from London for nursing (Clark, 1988). Indeed the rapid increase in rural wet-nursing of pauper and foundling children associated with the opening of the London Foundling Hospital in 1741 and Hanway’s Act led to a decline in the status of rural wet-nursing from the mid-eighteenth century. This may have made the practice increasingly unattractive to wealthier families, and fuelled a shift to retaining children in London. In this scenario the virtual unanimity amongst medical writers on the virtues of maternal breastfeeding, Enlightenment critiques of wet-nursing, and the cost and inconvenience associated with live-in nurses, may finally have persuaded many women to breastfeed.

Similar forces may have been at work in at least some other cities in north-western Europe. In Paris it has been estimated that half of infants were wet-nursed in the mid-eighteenth century, mostly in the countryside, but also that maternal breastfeeding had increased by the end of the century (Sussman, 1975, 1982, p. 22). Wet-nursing bureaux were in operation in Paris from the seventeenth century, but Sussman argued that greater official regulation of wet-nursing from the 1760s may have motivated high-status Parisian families to adopt maternal breastfeeding initially to avoid the imposition of such policing (Sussman, 1982, p. 32). Other French towns were less successful in instituting similar regulatory measures, and wet-nursing continued unabated. In Stockholm wet-nursing came under official regulation in 1757 with compulsory inspection and certification of wet-nurses, but it is not yet clear what impact this had on demand for or supply of wet-nurses (Hedenborg, 2001).

In addition to forces mitigating against wet-nursing it is also possible that maternal breastfeeding was adopted consciously to regulate fertility. It has been argued that the contraceptive effects of lactactional amenorrhoea were widely appreciated, and there are several eighteenth-century examples of high-status women who were urged not to breastfeed, in order to increase their fertility, and the chance of producing an heir (Fildes, 1986, pp. 108–109; Trumbach, 1978, p. 170). Conversely, a number of medical advocates of maternal breastfeeding emphasised the virtues of breastfeeding in helping to space births (Fildes, 1986, pp. 108–109; McLaren, 1985). Thus it is possible that the resort to maternal breastfeeding amongst higher-status women could have been driven by some new impetus to control their fertility in the late eighteenth century. Importantly however, the changes in birth intervals indicated convergence to a long-standing societal norm of protracted breast-feeding, rather than some culturally novel behaviour. Our data do not provide any evidence that higher-status families developed new motives or methods for controlling family size on a scale that might indicate an incipient fertility transition.

Our evidence corroborates Landers’ findings regarding trends in infant mortality in eighteenth century London, but also confirms that the Quaker sample was more representative of wealthy than of all families in the capital. In our sample mortality fell in all social groups across the late eighteenth and early nineteenth centuries, and improvements were most pronounced in the first month of life. However neonatal mortality was highest, and gains greatest, in the wealthier groups in our sample, and this was consistent with a shift to longer birth intervals and higher prevalence of maternal breastfeeding in these groups. Like Landers we found a puzzling persistence of a summer peak in neonatal mortality, a phenomenon we speculated might be due to hand-feeding in preference to colostrum in the first days after birth. In our sample improvements in mortality after the first month of life were confined to the period after 1795, except in wealthier families, where post-neonatal mortality declined in the last quarter of the eighteenth century as was the case amongst London Quakers.

It is particularly unfortunate that we could not pursue status differences in demographic behaviours into the period after 1800, when the effects of vaccination and increased maternal breastfeeding on mortality were most profound. By the first decade of the nineteenth century birth intervals in the sample as a whole had converged to the pattern of poorer women in the eighteenth century (Figure 2), and the relatively small differences between mortality of pauper and non-pauper infants suggest that there may have been substantial convergence in mortality also.

The lack of any advantage of wealth to survival in early life is consistent with the limited high-quality evidence available for pre-transitional populations (e.g. Bengtsson & van Poppel, 2011), and with what is known about urban environments and cultures of infant care in the eighteenth century. Even once breastfeeding habits appeared to have converged between high and lower-status groups, the removal of a marked disadvantage to newborns of wealthier families did not result in the emergence of a survival advantage to these infants. Rather it seems likely that breastfeeding provided a widespread, indiscriminate advantage to poor and rich infants alike, especially in the first month of life. These patterns are consistent with Reid’s finding that environment was a key determinant of infant mortality rates in late nineteenth century England. Although national data indicated a strong social gradient to infant mortality, infant mortality showed little variation by social class within local areas, indicating that the national patterns were a function of the sorting of social classes into different types of settlements and disease environments, rather than a direct effect of wealth or social class on household characteristics (Reid, 1997).

In later infancy and the second year of life mortality was dominated by infectious diseases, including smallpox, measles, scarlet fever, whooping cough and other childhood diseases, as well as diarrhoeal diseases associated with weaning. There were no cures for these diseases, and only smallpox could be prevented by artificial immunisation. While better nutrition, heating and clothing would be expected to have provided an advantage to wealthier infants, in our sample the lack of a marked disadvantage to paupers, whose families were least able to provide these amenities, after 1794 suggest that these factors were not major determinants of survival in early childhood. Rather it is probable that the severity of the metropolitan disease environment overwhelmed these factors to a great extent. The high densities of housing and the close residential proximity of rich and poor meant that all young children were exposed to epidemic diseases and the hazards of contamination of food and drink as a consequence of unclean water and inadequate disposal of sewage. The most important killer of young children, smallpox, did not discriminate by nutritional status, and was sufficiently lethal to kill adults as well as children. Wealthy parents may have chosen to inoculate their children, and this probably accounted for much of the mortality advantage associated with wealth in the period 1752–94 (Figure 4(g,h)). However this advantage was probably very substantially diminished with the very widespread acceptance of and access to vaccination after 1800.^10^ These complex and unstable patterns of socioeconomic differentials in survival of young children serve to illustrate the historical contingency of the relationships between health and wealth (Clouston, Rubin, Phelan, & Link, 2016).

Our study focussed on London in a period of very high but declining mortality. A key question is whether the social variations in infant-feeding practices evident in London extended to other British towns. It seems likely from diaries and documentary evidence that wet-nursing and other alternatives to breastfeeding were common to high-status families across the country before the nineteenth century (Fildes, 1988a, pp. 79–81). However evidence for large-scale wet-nursing and hand-feeding of infants outside the capital is scant. McLaren (1979) identified private nurse children from both Oxford and London in the Buckinghamshire parish of Chesham, and Sharpe argued that wet-nursing of local (often poor) infants was a common occupation in the Devon parish of Colyton (Sharpe, 2002, pp. 254–6). In Scotland the poll tax returns of the late seventeenth century indicate widespread wet-nursing of infants, and a pronounced social gradient to the practice, in Edinburgh (Marshall, 1988) as well as smaller towns (Desbrisay, 1997).

More research is required to establish whether low rates of maternal breastfeeding contributed to the excessive mortality of north-west European towns and cities before the nineteenth century. However the impact of smallpox vaccination on urban mortality was profound and must have made a major contribution to the ubiquitous improvements in urban mortality in the period 1800–20.

Although we have emphasised the importance of infant-feeding practices and smallpox immunisation, these cannot account for the improvements in especially neonatal mortality in poorer families (Figure 4(a–c)). Breastfeeding rates seem to have been reasonably high in poorer families across the period. Smallpox was not a major cause of death in the first month of life (due to protection by maternal antibodies acquired *in utero*). The main explanations offered for the falls in neonatal mortality at the national level is improvements in maternal health as a consequence of improved nutrition or lower levels of infectious disease exposure or susceptibility (Woods, 2009; Wrigley et al., 1997, pp. 317–318; Wrigley, 1998). Improvements in maternal nutrition would be expected to have the greatest effects on poorer women. However there was no evidence in our study that the health or nutritional status of poorer women was worse than that of wealthier women to an extent that might have compromised their fecundity,^11^ exerted deleterious *in utero* effects on their infants or rendered them unable to supply sufficient breast milk. Infants of poor families did not experience higher rates of mortality in the first week of life, when insults incurred *in utero* find their greatest expression (data not shown). Therefore the drivers of improvements in neonatal mortality at least were probably relatively egalitarian in effect. On balance our results are more consistent with widespread changes in maternal exposure to infectious diseases than with changes in nutritional status, since the latter would be expected to have been more socially selective in effect.

In conclusion the evidence presented here demonstrates the important contribution of urban cultures of infant feeding and childcare to excessive infant mortality rates in London and probably other English towns in the eighteenth century. Cultural change therefore probably played a significant role in the early stages of the mortality transition in English towns, together with medical innovations with regard to smallpox prevention. These factors may also have been important in driving mortality decline in other towns of north-western Europe. However our study also indicated the importance of other influences that were particularly key to improvements in survival of urban neonates, and these remain unidentified.

## Figures and Tables

**Figure A1.**
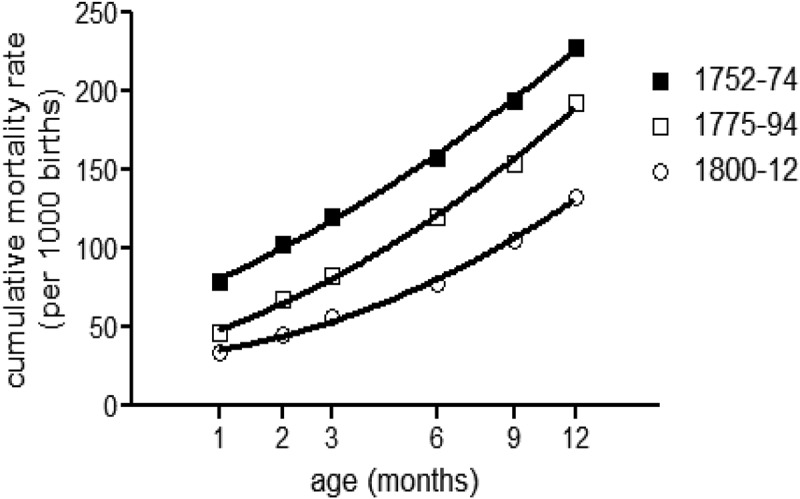
Unadjusted cumulative mortality over the first year of life. *Notes*: Curves fitted with second order polynomial equations to estimate endogenous mortality. Note log transformation of x-axis.*Source*: St. Martin in the Fields family reconstitution.

**Figure A2.**
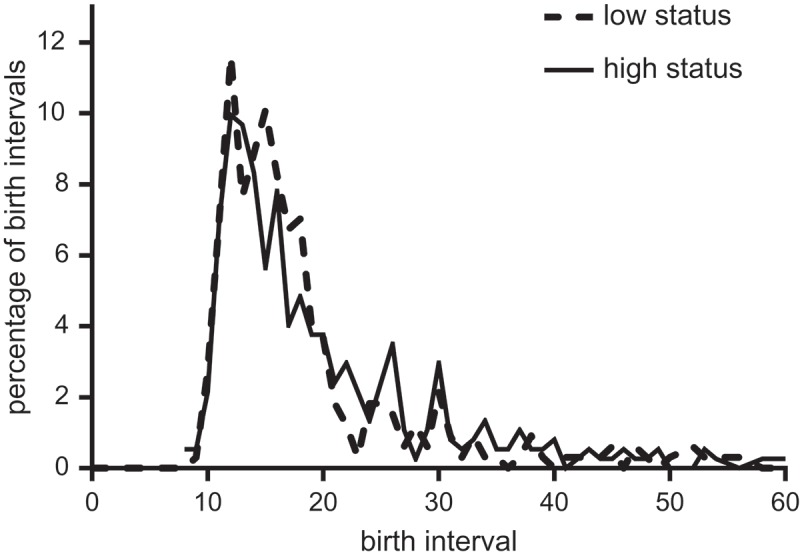
Distribution of birth intervals following the death of an infant in the first two months of life, by familial wealth, 1752–1794. *Notes*: P value from Kolmogorov-Smirnov exact test for equality of distributions of birth intervals = 0.139.*Source*: St. Martin in the Fields family reconstitution.

## Notes

1. See Appendix 1 for evidence that this bias did not inflate differences in birth intervals and mortality by wealth, but may have caused an *underestimation* of wealth effects.

2. The Westminster Historical Database project only transcribed entries for male ratepayers.

3. We excluded birth intervals of longer than 60 months, because of the risk that longer intervals concealed an unobserved residential move.

4. This lengthening of birth intervals was not an artefact of an increased failure to capture some birth events (resulting in the inclusion of some birth intervals between births a and c because intervening birth b was missed), because there was no lengthening of the ‘tail’ of long birth intervals, and birth capture was fairly complete (Davenport, 2016). An alternative explanation for both the differences in birth intervals by social status and the shift is that these patterns are artefacts of differences in the ages of women comprising each group. Higher status women may have married younger. Younger women are more fecund on average and therefore may have had shorter birth intervals than older women. We don’t know the ages of the women in our sample. However a crude test for differences in fecundity, described in Appendix 4, indicated no differences between status groups.

5. See Appendix 1 for evidence that these results were not an artefact of our sample composition.

6. Y = a*X + b*Z where Y is the distribution of birth intervals in the case where the fate of the first child is unknown, X the distribution in the case where the first child died, Z is the distribution of birth intervals where the first child survived infancy, and a + b = 1. The proportion of unexpectedly short birth intervals in the case where the fate of the infant whose birth opened the interval was unknown (a) was calculated for the combined wealth groups (0 plus 1, and 2 plus 3). These were then converted to an estimate of numbers of missing children in each individual wealth category on the assumption that the proportion missing of infants of unknown fate took the same value (a) for each group in the combined wealth group: missing infants in sample = a*Y/(X + Y + Z).

7. To the extent that this method can be used to estimate levels of extra-parochial nursing, it will provide minimum estimates, because some children who died at nurse may have been returned to St. Martin’s for burial, and would not appear in the ‘unknown fate’ category.

8. But see also Clark, 1987, pp. 13–15, regarding the likelihood of private baptism in order to expedite delivery of the child to its nurse for feeding.

9. It is plausible that infant mortality was higher amongst London Quakers than the St. Martin’s reconstitution sample because very early weaning or absence of maternal breastfeeding was apparently the norm in Landers’ sample of Quakers in the mid-eighteenth century, and so infants were at very high risk compared with the St Martin’s sample where infant-feeding practices were more mixed.

10. Sundin noted a similar pattern in the Swedish town of Linköping, where rates of smallpox were lower amongst middle-class families before the first mass vaccination of infants in 1802 (Sundin, 1995, p. 133). Perrenoud attributed the very large differences in child mortality in Geneva in large part to the influences of crowding and family size on smallpox mortality, and vaccination may have contributed to the closing of the gap between middle class and poor families by the nineteenth century (Schumacher & Oris, 2011).

11. See Appendix 4.

## Appendix 1. Event history analysis

The reconstitution process used is set out in Davenport (2016), where the apparent completeness of birth capture is demonstrated. The percentage distribution of births per family is given in Figure 3 of Davenport (2016). Burials by age and sex are set out in Table A1. Unadjusted mortality rates were calculated using survival analyses, performed using Stata software version 14.1. Families were deemed in observation from the date of the parents’ marriage, or from the birth of the first child in observation if this occurred within three months of the baptism (otherwise baptism). ‘First’ births (that is first in observation, but not necessarily first to the couple) constituted a relatively high proportion of all births in the sample, because only a fraction of most birth histories was observed. It was necessary to use birth date rather than baptism date as the date of entry to analysis for these first births because if these infants had entered only at baptism then the often substantial lag between birth and baptism would have resulted in the exclusion of a high proportion of those who survived the first month from the population at risk in the first month of life (because they were not yet registered as baptised). Similarly, those most at risk (infants baptised early or who died before baptism and were assigned a birth date) would have been retained in the sample, which would have resulted in a very substantial overestimation of mortality in early infancy. The assumption made in this case was that families baptising within three months of birth had been resident in the parish since the birth in question. This was felt to be justified in the first three months after birth on the basis of migration rates estimated from modelling of burial-baptism linkages by age (Davenport, 2016) but was less tenable with longer birth-baptism intervals. There was no significant difference in the distributions of birth-baptism lags between private and public baptisms or by social status so this procedure should not have biased estimates of mortality. Where a burial with only a dummy birth was the first event in a family then this was excluded unless the death occurred in the first three months of life (for the reasons given above). Families exited observation at the date of the last observed baptism to the family, and the last child was excluded from analysis. Families were only deemed to be in observation so long as they recorded events at the same street address (Davenport, 2016).

**Table A1.** Burials by age and sex included in analyses, 1752–1812.

Age (days)	Female	Male	Unknown	Total
0–6	28	43	94	165
7–29	31	37	2	70
30–60	188	196	11	395
61–91	98	114	3	215
92–182	56	85	2	143
183–364	136	191	3	330
364–729	152	173	2	327
Total	689	839	117	1645

*Notes*: Sex was determined from the forenames of children recorded in the sextons’ burial books. Some forenames were ambiguous and were therefore classified as ‘Unknown’. Stillborn infants are excluded.

*Source*: St. Martin in the Fields family reconstitution.

The use of baptisms to track whether families remained in observation, and the requirement that families remained at the same street address at successive baptisms, introduced several potential biases into our comparisons of birth intervals and mortality by wealth. This was because birth intervals themselves influenced the probability of being included in the reconstitution sample, with families with longer birth intervals being more likely to be excluded from the sample because they moved between baptisms. This could have biased our comparison of birth intervals by wealth, because poorer families were characterised by higher residential mobility than rich families. When we compared addresses for infants who died within a year of baptism, 30% of pauper infants born outside the workhouse had changed address with twelve months, compared with 26% for those paying modest burial fees, and 19% for those paying 8 s 6 d or more for burial (overwhelmingly higher status families – see Table 3, and Davenport, 2016, Table 4).

If longer birth intervals were missed because families changed residence between births, then observed birth intervals would be predicted to be shorter amongst the poor, because they were more likely to change address between births. However, birth intervals displayed a wealth gradient that was in the opposite direction to the wealth gradient in residential mobility (see main text). That is, families with the highest residential stability displayed the shortest birth intervals, and the most mobile families (paupers) the longest intervals. Our data thus do not suggest a strong effect of mobility on our measures of birth intervals, although it is possible that higher residential mobility amongst poor families caused us to *underestimate* birth intervals in poorer families, and therefore to underestimate the differences in birth intervals by wealth. It was also possible that this problem biased estimates of mortality. Infant deaths were associated with shorter birth intervals (because of the associated cessation of breast-feeding), and therefore families with higher mortality were more likely to be included in the reconstitution sample. Again however, the effect of this bias would be to cause an underestimation of differences in mortality by wealth, because the wealthier and more residentially stable families had the highest infant mortality rates, as demonstrated below.

A second possible source of bias introduced by residential mobility was the seasonality of residence in London amongst the wealthy. Many affluent families left London during the summer and parliamentary and legal court recesses, and returned in the autumn or winter for the ‘London Season’ (see Schwarz, 1992, pp. 105–107 for a discussion of the shifting seasonality of the Season). However we could detect no seasonality of baptisms amongst wealthy families that would indicate that our sample included many families who were absent for the warmer part of the year.

## Appendix 2. Biometric analysis of mortality in the first year of life

The classic test for under-registration of burials is the Bourgeois-Pichat or biometric test. Bourgeois-Pichat (1952) argued that infant mortality displayed a consistent age pattern regardless of the actual level of mortality, such that if plotted on an appropriate log scale the cumulative rate of mortality over the first year approximated a linear function. The slope of the fitted line was interpreted to represent the accumulation of deaths due to ‘exogenous’ causes, mainly infectious diseases. Where the fitted line crossed the x-axis, at birth, this value gave a measure of the ‘endogenous’ component of mortality, those deaths attributable to causes arising *in utero*, during the birth process or deriving from genetic factors, and includes major causes such as prematurity and low birth weight as well as infections acquired during birth such as neonatal tetanus and early onset sepsis. The technique could also be used to detect under-registration of early infant deaths in the case where the constant was too small to be plausible or the linear fit was poor. Subsequent work on historical populations has demonstrated that non-linear fits to cumulative infant mortality are more appropriate than linear fits under certain scenarios where infectious disease mortality was very high especially in the second half of the first year of life as a consequence of early weaning or very high exposure to disease in the context of waning maternally derived immunity (Knodel & Kintner, 1977; Woods, 2000).

Figure A1 shows biometric plots of infant mortality for all reconstitution families for three periods. For all periods non-linear equations (second-order polynomials) provided a better fit than linear equations, consistent with the extreme infectious disease environment of London in this period. Endogenous mortality calculated in this way was comparable to rates amongst London Quakers and the national sample (not shown). However these endogenous mortality rates were rather close to total neonatal mortality rates, implying that almost all mortality in the first month of life was concentrated near birth. While this was plausible for the national, largely rural sample, it was in contrast to the pattern amongst London Quakers, where mortality was also high later in the first month of life. The pattern in St. Martin’s of high endogenous relative to total neonatal mortality suggested that any under-registration of burials was not confined to the early neonatal period. An alternative more discriminating method of detecting omission of early neonatal deaths uses the proportions of twin births in the reconstitution sample. Twins were at much higher risk of death than singleton infants in the first days after birth, and were therefore at higher risk of dying unregistered. However twins were not under-represented in the reconstitution sample, suggesting that the capture of very early deaths was relatively complete (Davenport, 2016). Therefore the suspiciously low rates of neonatal mortality in St. Martin’s were likely to be a function of under-registration not of very early infant deaths, but of burials of older neonates (and by implication older infants).

## Appendix 3. Adjustments to mortality rates for missing infants

Mortality rates were calculated using product-limit life tables that recalculate probabilities of survival every time a death or an exit from observation (right-censoring) occurred. Unadjusted mortality rates were calculated using individual-level data, however to adjust mortality rates for missing infants it was necessary to apply adjustments to the life table as a whole, because individual infants could not be identified as missing.

To adjust for missing infants on the assumption that they were sent out of London to be wet-nursed we assumed that they left the sample at age 7 days, and removed them from the denominator of mortality rates at all successive ages. This had the effect of reducing the population at risk but leaving the burial count unchanged. We had no further knowledge of the death rates of infants at nurse, and so this method essentially assumed that the mortality rates amongst those omitted were similar to those remaining in the sample and so they could be omitted without biasing mortality.

To adjust for missing infants on the assumption that they remained in the parish but their deaths went unobserved, then it was necessary to add back all the missing infants as burials. We assumed that the age at death of missing exported burials was the same as that of known burials in the sample, except in the first week of life (where capture of burials appeared fairly complete, and where burial exports were rare: Davenport, 2016). The proportion of burials exported was fairly constant at ages above 1 week. The number of infants calculated as missing (Table 5) was therefore added back into the life table as deaths in proportion to recorded deaths in the life table at each age at ages 7–364 days.

The assumption that infant mortality was underestimated due to unobserved burial export also applied to mortality at older ages. However the method used to estimate the numbers of missing infants was only applicable to the first year of life (since it relied on the effects of breastfeeding on birth intervals, and so only captured missing deaths at ages before a high proportion of the sample was weaned). In order to inflate mortality in the second year of life for the effects of unobserved burial export it was assumed that proportion of burials missing was the same in the second year of life as in the first.^12^ This problem did not apply to the calculation of mortality at age one in the case where missing infants were assumed at nurse, because the adjustment of the population at risk applied at age 7 days resulted in an automatic adjustment of the mortality rates at all subsequent ages. This latter adjustment assumed that infants sent to nurse did not return in the second year of life, an assumption borne out by the rules and practices of the Foundling Hospital and numerous anecdotal accounts of private nursing that indicate that infants sent away to nurse remained at nurse for several years in the eighteenth century (see also Newton, 2011). The number of censored observations was adjusted in both scenarios to prevent missing infants from exiting twice.

Because adjustments were necessarily made to each social group in aggregate and did not apply to individual-level records it was not possible to compare adjusted mortality rates statistically using proportional hazards-type models (although we could have constructed synthetic cohorts for this purpose). Similarly, it was not possible to investigate the effects of individual-level attributes such as street address, duration of residence or birth order (in cases where marriage date was known).

## Appendix 4. Maternal health and fecundability

The analysis of birth intervals also allowed us to probe the issue of whether there were differences in fecundity, or biological capacity to conceive, by wealth. We couldn’t measure fecundity directly, but could use the concept of fecundability to estimate differences and trends in fecundity. Fecundability is defined as the monthly probability of conception, in women at risk of conception (for our purposes, married, of reproductive age, and not breastfeeding). It depends on coital frequency and on parental fecundity (potential for conception). We could measure fecundability in our sample using birth intervals, but could not separate out its components. Our method compared the time to conception following the early death of an infant.

Taking the distributions of birth intervals following the death of an infant, the slower rise and later peak of the distribution in the poorer half of the sample suggested superficially some delay in conception following the death of an infant in this group compared with wealthier women (Figure A2). However, infant deaths were distributed more evenly across the first year of life in the poorer group, and breastfeeding was more common, factors that would act to prolong average birth intervals in this group. To remove the effects of these inter-group differences we considered only the distribution of times to next birth following the death of an infant in the first two months after birth (measuring the interval between death and the next birth, rather than between the birth of the first infant and the subsequent birth). This removed effects of differences in the distributions of age at infant death, and minimised the effects of breastfeeding, since women who breastfed could have done so for at most two months compared with women who never initiated breastfeeding. When the data were compared in this way then there was no evidence for any greater delay in conception amongst the poorer group (Figure A2). To the extent that the rapidity of conception following the early death of an infant could be interpreted as a measure of fecundity, then there was no evidence that poorer women were less fecund than those of wealthier families.
